# Navigating Undergraduate Medical Education: The Impact of Enhanced Mentorship Pairing at a New Medical School

**DOI:** 10.7759/cureus.62789

**Published:** 2024-06-20

**Authors:** Shawn Izadi

**Affiliations:** 1 Surgery, Oregon Health & Science University, Portland, USA; 2 Medical Education, University of Texas Rio Grande Valley School of Medicine, Edinburg, USA

**Keywords:** mentorship programme, good mentor, peer mentorship, undergraduate and graduate medical education, mentorship program

## Abstract

Introduction

Transitioning into medical school is challenging, particularly in the first year, with a notable support gap. This study aimed to evaluate a mentorship program at a new medical school.

Methods

Initiated in 2017 at the University of Texas Rio Grande Valley School of Medicine, the mentorship program had two iterations: initial random pairings and subsequent formative pairings based on matching criteria. A mixed-methods approach assessed its effectiveness in supporting first-year students.

Results

Of 109 first-year students, 76% completed a 6-month survey. Both classes primarily had male mentees with varied interests in primary or specialty care. No significant demographic differences or benefits between 1:1 and 2:1 mentor-mentee pairings were found, though in-person communication was preferred in 1:1 pairings (p=0.036). While enhanced matching criteria improved perceived transitions (p=0.47) and academic performance (p=0.84), these did not reach statistical significance. However, it increased the frequency of communication (p=0.038).

Conclusion

The implementation of a peer-mentorship program at a new medical school demonstrates high engagement among first- and second-year medical students with perceived improvement in transition and academic performance. Although enhanced matching criteria led to more frequent communication, highlighting the significance of careful mentor-mentee pairings, they did not correlate with better transitions or academic outcomes. This indicates that while these criteria are valuable, they are less crucial than simply having a mentorship program in place.

## Introduction

Peer mentorship programs have gained increasing recognition and popularity in medical education as valuable tools for supporting students' transition into medical school and fostering their academic and professional development [1]. These programs typically involve pairing incoming or junior students with more experienced peers who serve as mentors, offering guidance, support, and advice throughout the undergraduate medical journey.

The transition into medical school is a critical period characterized by numerous challenges, including adapting to the rigorous academic curriculum, navigating the complexities of the healthcare environment, and managing personal and professional responsibilities [2]. Peer mentorship programs have emerged as effective interventions to address these challenges by providing a structured support system that complements formal academic resources and enhances students' sense of belonging and confidence with some programs seeing mean program satisfaction scores increase by 20% after the implementation of a peer mentorship program [1]. The effectiveness of peer mentorship programs lies in their ability to facilitate peer learning, role modeling, and social support among students [3]. By connecting mentees with mentors who have successfully navigated similar challenges, these programs create opportunities for knowledge exchange, skill development, and professional networking. Additionally, mentorship relationships can foster personal growth, resilience, and well-being among both mentors and mentees, contributing to a positive learning environment and promoting a culture of collaboration and mutual respect [4].

Despite the growing popularity of peer mentorship programs in medical education, there remains a need for evaluation and assessment of their impact and effectiveness. While anecdotal evidence and qualitative studies have highlighted the benefits of mentorship, both quantitative and qualitative research examining the implementation of mentorship programs at new medical schools is limited, particularly in their implementation [1]. Moreover, variations in program design, particularly in the way that students are paired with each other should be examined more thoroughly. In light of these considerations, this study aims to uniquely investigate the creation and implementation of a peer mentorship program between first-year and second-year medical students at an inaugural medical school.

## Materials and methods

Mentorship program overview

The medical student mentorship program was established in 2017 at the University of Texas Rio Grande Valley School of Medicine (UTRGV SOM). Initially from 2017 to 2018, the program relied on a random pairing system, where first-year students were matched with second-year students based solely on availability and logistical considerations.

From 2017 to 2018, the mentorship program successfully facilitated connections between first-year and second-year medical students, providing mentees with valuable insights into the medical school experience and offering mentors an opportunity to share their knowledge and experiences. After 2018, the mentorship program underwent an evolution in response to student feedback. Building upon the foundation established in the first year, the program transitioned to a more intentional matching process based on personality and interest surveys completed by both mentors and mentees. These surveys allowed participants to express their preferences, strengths, and areas of interest, enabling the program coordinators to facilitate more compatible and mutually beneficial mentor-mentee pairings.

Participants

This study received approval from the institutional review board (IRB) of the University of Texas Rio Grande Valley and was deemed exempt. Informed consent was obtained from all participants prior to their participation in the mentorship program and separately for the administered survey. Mentees in the study consisted of first-year medical students of the Class of 2021 at UTRGV SOM during the first iteration of the mentorship program followed by mentees from the Class of 2022 at UTRGV SOM during the second iteration of the mentorship program.

Incoming students posed to be mentees were presented with an “opt-out” approach and there were no students who chose not to participate. Additionally, mentors in the program were second-year medical students (Class of 2020 and Class of 2021, respectively) who volunteered to participate. There was no faculty involvement or pairings for this study purpose.

Procedure

For the first iteration of the mentorship program, the peer mentorship program was initiated at the beginning of the academic year (July 2017). Mentees were paired with their mentors randomly based on a random number generator.

The second iteration of the mentorship program was initiated at the beginning of the following academic year (July 2018). Mentees from the Class of 2022 were provided with an introduction survey that allowed them to include free-response answers to questions that explored hobbies, interests, and personality types (Appendix A). Simultaneously, second-year students from the Class of 2021 were recruited to serve as mentors through a survey process that included the same questions posed to mentees, including those surrounding hobbies, interests, and personality types. The matching of mentors and mentees was facilitated based on similarities between these hobbies, interests, and personality types from collected surveys.

Once mentorship pairs were established, mentors and mentees were provided with guidelines and resources to structure their mentorship interactions. These interactions encompassed various aspects such as academic support, career guidance, personal development, and social integration into the medical school community.

Data collection

Data for this study were collected at two distinct time points - 1 survey for each iteration of the mentorship program administered to mentees 6 months after the start of the respective academic year (December 2017 and December 2018). The survey instrument included a combination of Likert-type scales (7 levels including a neutral level) and open-ended questions designed to assess mentees' perceptions of the effectiveness and impact of the mentorship program (Appendix B).

The Likert-type scales measured mentees' satisfaction levels regarding different aspects of the mentorship experience, including mentor availability, communication effectiveness, and perceived benefits gained from the mentorship. Open-ended questions allowed mentees to provide qualitative feedback, share specific examples of helpful mentorship experiences, and offer suggestions for program improvement, including critical feedback.

Data analysis

Quantitative data obtained from the Likert-type scales were analyzed using descriptive statistics to calculate median scores and standard deviations and perform chi-squared comparisons. Findings from the data analysis were used to evaluate the effectiveness of the program, identify areas of strength, and pinpoint areas for potential improvement. Analyses were performed with Stata 18 (StataCorp. 2023. Stata Statistical Software: Release 18. College Station, TX: StataCorp LLC).

## Results

Participation and mentor/mentee characteristics

All 109 first-year medical students (55 for the Class of 2021, 54 for the Class of 22) chose to participate as mentees in their respective year’s mentorship program with no student choosing to opt-out. Seventy-one second-year medical students (32 for the Class of 2020 and 39 for the Class of 2021) chose to participate as mentors in their respective year’s mentorship program representing a 65% participation rate. Eighty-three mentees (76%) completed the six-month survey for their respective mentorship year.

The majority of mentees from the Class of 2021 were male (n=29, 53%) with a strong initial interest in primary care (n=36, 65%). Their mentors shared similar traits with the majority being male (n= 19, 59%) but with a stronger interest in specialty care with only 14 (44%) interested in primary care. The mentees from the Class of 2022 were majority male (n=28, 52%) with an almost even split between those who wanted to pursue primary care (n=26, 48%) compared to specialty care (n=28, 52%). Similarly, their mentors were majority male (n=21, 53%) with a similar split between those who wanted to pursue primary care (41%) compared to specialty care (n=23, 59%) (Table 1).

**Table 1:** Characteristics of mentees and mentors

	Class of 2021 Mentees (n=55)	Class of 2022 Mentees (n=54)	
Sex			
Male	29 (53%)	28 (52%)	p=0.93
Female	26 (47%)	26 (48%)	
Specialty Interest			
Primary Care	36 (65%)	26 (48%)	p=0.07
Subspeciality Care	19 (35%)	28 (52%)	
	Class of 2020 Mentors (n=32)	Class of 2021 Mentors (n=39)	
Sex			
Male	19 (59%)	21 (53%)	p=0.64
Female	13 (41%)	18 (47%)	
Specialty Interest			
Primary Care	14 (44%)	16 (41%)	p=0.82
Subspeciality Care	18 (56%)	23 (59%)	

There was no statistically significant difference in either cohort between mentors and mentees for either male and female ratio or primary care for subspeciality interest (p>0.05).

Comparison of mentor pairing structures

Fifty-five first-year medical students were matched with 32 second-year medical students during the first iteration of the program resulting in 9 1:1 pairs and 23 2:1 pairs. Fifty-four first-year medical students were matched with 39 upperclassmen mentors during the second iteration of the mentorship program resulting in 24 1:1 pairs and 15 2:1 pairs.

During the program’s first iteration when comparing the experiences of students in different mentor pairing structures, 7 out of 9 1:1 individuals (78%) in a 1:1 pairing reported at least somewhat agreeing that the program aided their transition into medical school, while 16 of 23 (70%) in a 2:1 pairing reported at least somewhat agreeing that the program aided their transition into medical school (p=0.85). Eight of 9 1:1 individuals (89%) chose to use in-person as their primary mode of interaction compared to only 5 of 23 (22%) in a 2:1 pairing (p=0.036).

During the program’s second iteration when comparing the experiences of students in different mentor pairing structures, 10 out of 11 individuals (91%) in a 1:1 pairing reported at least somewhat agreeing that the program aided their transition into medical school, while 19 out of 23 individuals (82%) in a 2:1 pairing reported at least somewhat agreeing that the program aided their transition into medical school (p=0.41). Eight of 11 1:1 individual (73%) chose to use in-person as their primary mode of interaction compared to only 4 of 23 (17%) in a 2:1 pairing (p=0.038).

Chi-square analysis in either iteration demonstrated no statistically significant difference in perceived benefit between 1:1 and 2:1 mentor relationship in either program iteration but there was a significant difference in the primary mode of communication favoring in-person meetings for 1:1 pairings.

Impact of matching criteria

**Figure 1:**
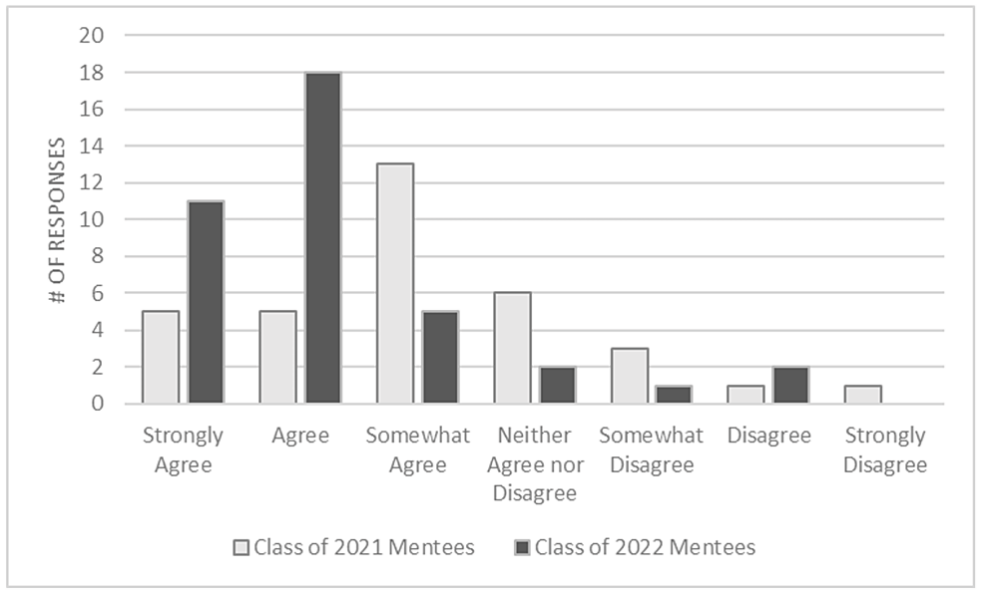
Comparison of perceived aid in transition to medical school by year

In the first iteration of the program, 23 of 34 (68%) reported at least somewhat agreeing that the program aided their transition into medical school with only 5 of 34 (15%) strongly agreeing with that assessment. This was improved upon in the second iteration of the program with the introduction of more formative pairings as 34 of 39 (87%) reported at least somewhat agreeing that the program aided their transition into medical school with 11 of 39 (28%) strongly agreeing (p=0.47) (Figure 1). 

**Figure 2:**
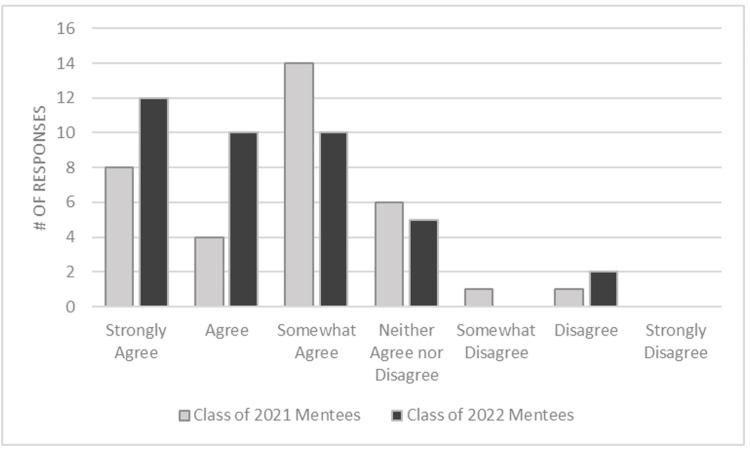
Comparison of perceived benefit to academic performance by year

Similarly in the first iteration of the program, 26 of 34 (76%) reported at least somewhat agreeing that the program positively affected their academic performance with 8 of 34 (24%) strongly agreeing with that assessment. This was improved upon in the second iteration of the program with the introduction of more formative pairings as 32 of 39 (82%) reported at least somewhat agreeing that the program positively affected their academic performance with 12 of 39 (31%) strongly agreeing (p=0.84) (Figure 2).

In the first iteration of the program, the majority of mentees (n=18, 53%) stated that they would communicate with their mentor only once per month compared to the majority of mentees in the second iteration of the program (n=21, 54%) stating they would communicate with their mentor once per week.

## Discussion

The findings of this study suggest that the peer mentorship program implemented between first-year and second-year medical students at our inaugural institution was generally effective in aiding the transition into medical school and positively impacting academic performance. The high percentage of participants who reported feeling supported and benefited from the program aligns with previous research highlighting the importance of peer support in medical education [4]. The positive trends observed in various aspects of mentorship, including adjustment to the local area and increased social support, underscore the multifaceted benefits of mentorship in medical training. In light of this, many higher education programs utilize these types of programs to help provide an additional tool for students to build academic and peer-support success [3].

A unique aspect of our study is the dynamic transition that took place between the first and second year of the program’s matching process. In its infancy, the peer-mentorship program used a random sorting process to create the mentor-mentee pairings. Although feedback was generally positive, the initial round of surveying demonstrated significant areas for improvement, especially in the realm of frequency of communication and perceived aid in the transition to medical school. Although surveys demonstrated that a majority of students felt the program aided in their transition into medical school, very few mentees stated that they reached out to their mentor multiple times for advice. This information led us to believe that there were large areas for improvement, especially surrounding mentor-mentee matching to ensure the most positive experience for everyone involved. This is highlighted by Cesa and Fraser et al. as they describe how randomly assigning students to a mentor typically is associated with the lowest degree of success [5].

In the second iteration of the peer-mentorship program, the unique change was to match students based on traits that the mentee deemed most important to them (i.e. gender, personality traits, specialty interests, etc.) similar to what others have described [6-8]. The adoption of a tailored matching approach aimed to enhance the quality and effectiveness of mentorship relationships by fostering greater rapport, understanding, and alignment between mentors and mentees. By pairing individuals with shared interests, complementary personalities, and similar career aspirations, the program sought to create a supportive and enriching mentorship environment conducive to personal and professional growth. With this adjustment, we witnessed improvements in almost every aspect of the surveying despite minimal adjustments to other areas of the program. Mentees reported more frequent communication with their mentors and reported higher scores on their surveys for both perceived aid in the transition to medical school and positively affected academic performance. Despite these not achieving statistical significance in our analysis, likely due to low power, the benefits of a structured and more formative matching process for mentees and mentors cannot be underscored.

Comparison of mentor pairing structures

Our study did not find a significant difference in perceived benefit between 1:1 and 2:1 mentor pairing structures. While there is a paucity of literature that delves into the effects of different mentor-mentee ratios, our results indicate that both structures can be equally effective in supporting mentees' transition into medical school [3]. This finding is particularly advantageous for institutions with resource constraints. By demonstrating that both mentorship structures yield comparable outcomes, our study implies that institutions can adapt their mentorship programs to fit their available resources without compromising their effectiveness. This flexibility in mentor-to-mentee ratios allows institutions to allocate resources more efficiently and accommodate a larger number of mentees within their programs. Moreover, it encourages the exploration of alternative mentorship models that may better suit the specific needs and dynamics of individual institutions or student cohorts. Interestingly, our study highlighted that those with a 2:1 pairing were more likely to engage in phone/text conversations compared to in-person for the 1:1 pairings. This makes logical sense due to the constraints of being a mentor for two mentees.

Furthermore, our findings underscore the importance of considering various factors beyond the mentor-to-mentee ratio when designing mentorship programs. While personalized attention remains a valuable aspect of mentorship, other factors such as mentorship quality, compatibility between mentors and mentees, and the structure of program activities may also significantly influence mentees' experiences and outcomes.

Impact of matching criteria

The incorporation of matching criteria, including factors such as gender, personality traits, and specialty interests, into mentor-mentee pairings has demonstrated substantial advantages over random pairings within our program [5]. This finding resonates with prior research highlighting the significance of compatibility and shared interests in mentorship relationships [1,8,9]. By leveraging these criteria to match mentors and mentees, our program achieved notable improvements in the overall benefit experienced by participants.

One of the key outcomes of our study was the significant increase in the proportion of participants reporting that the mentorship program aided their transition into medical school. In the first iteration, 68% of participants agreed that the program contributed to their transition, with only 15% strongly agreeing. However, in the second iteration, with enhanced matching criteria, this proportion rose to 87%, with 28% strongly agreeing. Although the difference in perceived benefit between the two iterations was not statistically significant (p=0.47), the substantial increase in the number of participants strongly agreeing suggests a meaningful improvement in the program's effectiveness.

Similarly, our findings revealed a positive impact on academic performance, with more participants in the second iteration reporting that the program positively affected their academic performance compared to the first iteration. In the first iteration, 76% of participants agreed that the program positively affected their academic performance, with 24% strongly agreeing. In contrast, in the second iteration, 82% agreed, with 31% strongly agreeing. Although the difference in perceived benefit between the two iterations was not statistically significant (p=0.84), the increase in the proportion of participants strongly agreeing suggests a potential enhancement in academic outcomes associated with the more formative pairing approach. Although not extracted from our survey, other groups have demonstrated the psychological benefit of these pairings that also aid in improved academic performance [10].

Furthermore, our study highlighted a shift in communication frequency between mentees and mentors between the two iterations. In the first iteration, the majority of mentees communicated with their mentors only once per month. However, in the second iteration, the majority communicated once per week, indicating a more frequent and potentially more beneficial interaction pattern. Similarly, previously successful mentorship programs have harnessed the power of increased communication, particularly within the minority population to enhance the mentor-mentee relationship [11]. This change underscores the importance of effective communication in mentorship relationships and suggests that enhanced matching criteria may facilitate more meaningful and regular interactions between mentors and mentees.

Limitations and future directions

While our study provides valuable insights into the effectiveness of a peer mentorship program for medical school, several limitations should be acknowledged. First, the survey data relied on self-reported measures, which may be subject to response bias and social desirability effects. Additionally, the relatively small sample size and single-institution study design limit the generalizability of our findings and limit our power to detect small yet notable statistical differences. Despite this, there is uniqueness in our study being implemented at a new medical school and providing a framework for how others can similarly institute a mentorship program. Also, it must be acknowledged that there was a transition from random pairings in the previous iteration of the program to a more structured pairing system in the current, studied format. Unfortunately, surveys were not conducted for the original iteration of the mentorship program, thereby losing significant comparison data. But despite this, the resoundingly positive survey data from the current iteration speaks to the nature and quality of the current mentor-mentee relationships. Future research could explore the long-term effects of the mentorship program on mentees' academic and professional development, as well as investigate additional factors influencing mentorship outcomes such as mentor-mentee rapport and mentor training.

## Conclusions

The implementation of a peer mentorship program at a new medical school is a promising tool to aid the transition and maintain the well-being of first-year students during the critical start of their undergraduate medical education. Although enhanced matching criteria led to more frequent communication, highlighting the significance of careful mentor-mentee pairings, they did not correlate with better transitions or perceived academic outcomes. This indicates that while these criteria are valuable, they are less crucial than simply having a mentorship program in place. Nevertheless, comprehensive, long-term studies involving all students and their mentors are necessary to assess the effectiveness of the program.

## Figures and Tables

**Figure 3:**
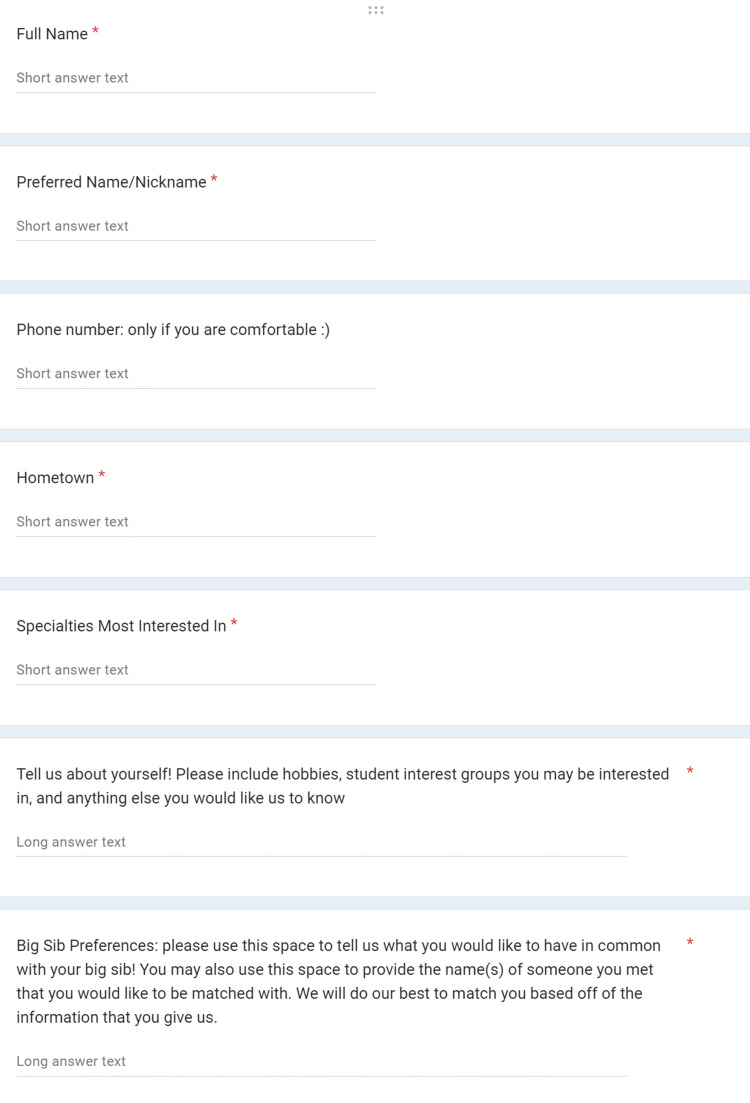
Mentee sign-up survey

**Figure 4:**
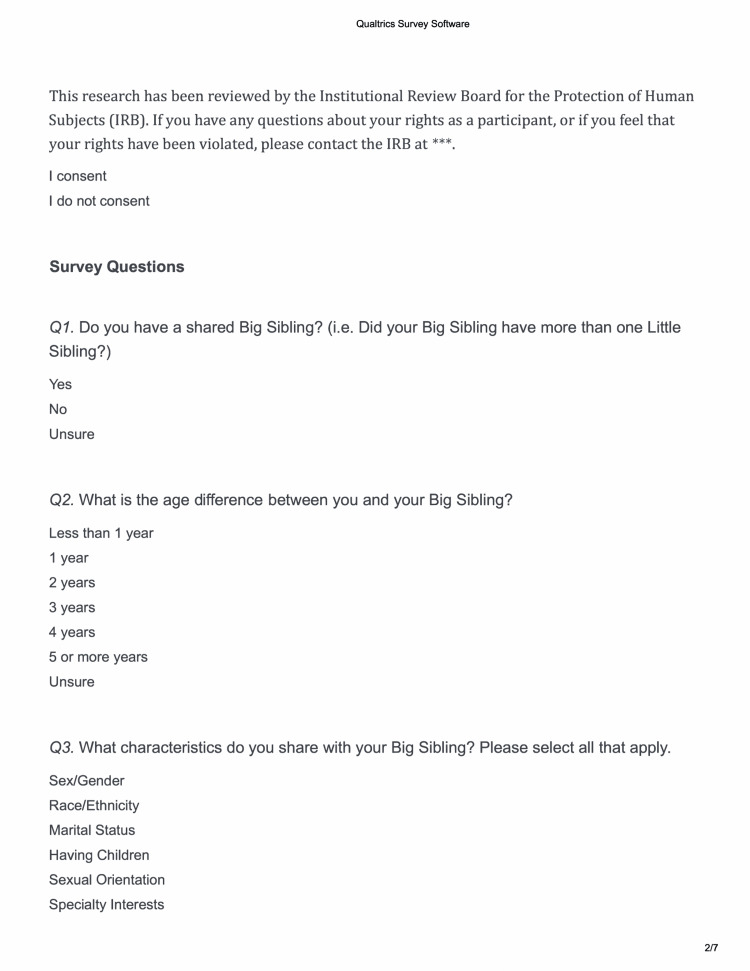
Six-month mentee evaluation survey (page 1)

**Figure 5:**
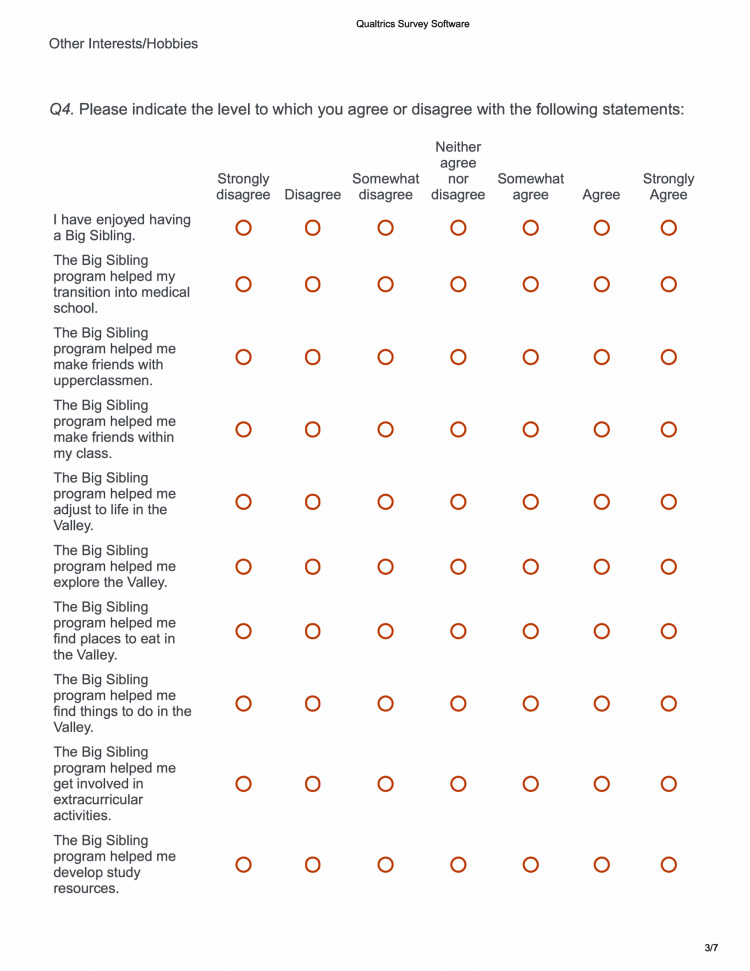
Six-month mentee evaluation survey (page 2)

**Figure 6:**
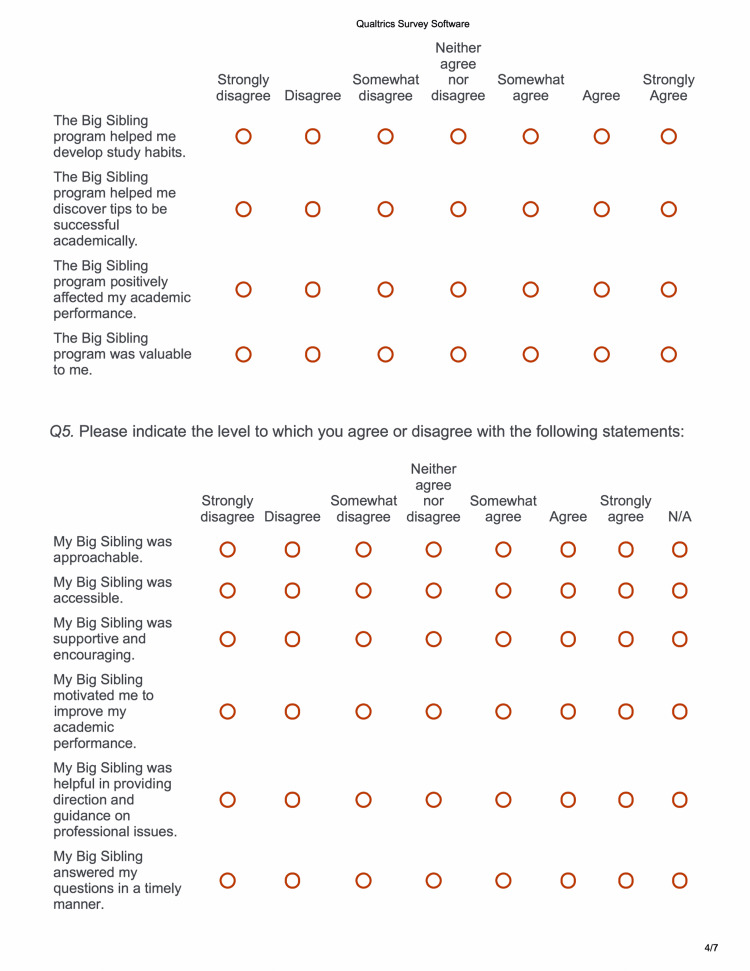
Six-month mentee evaluation survey (page 3)

**Figure 7:**
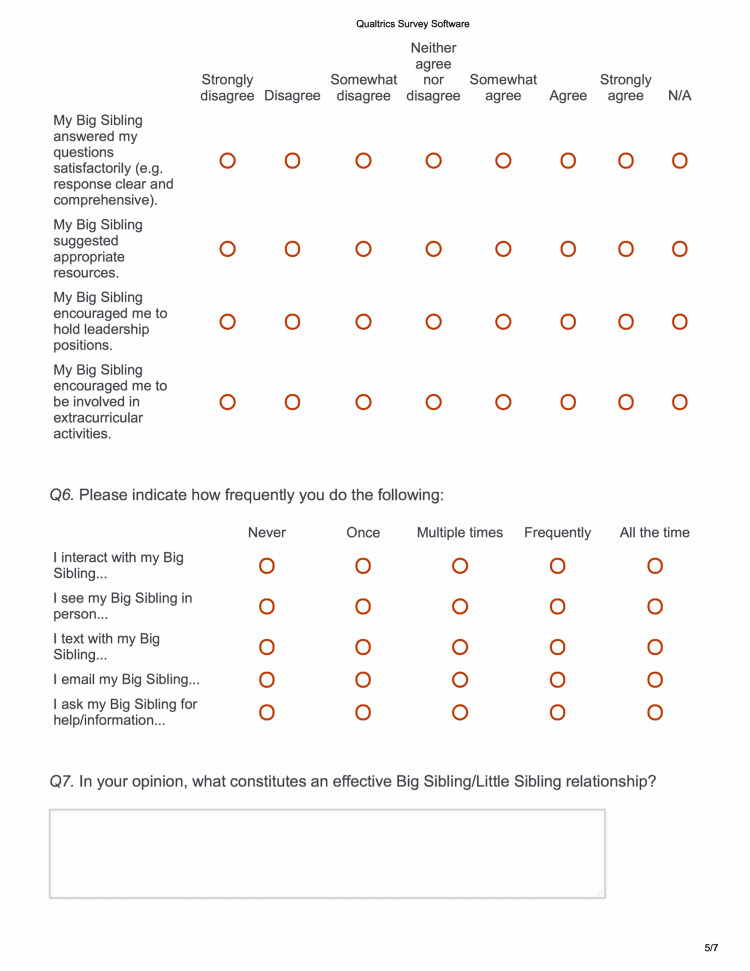
Six-month mentee evaluation survey (page 4)

**Figure 8:**
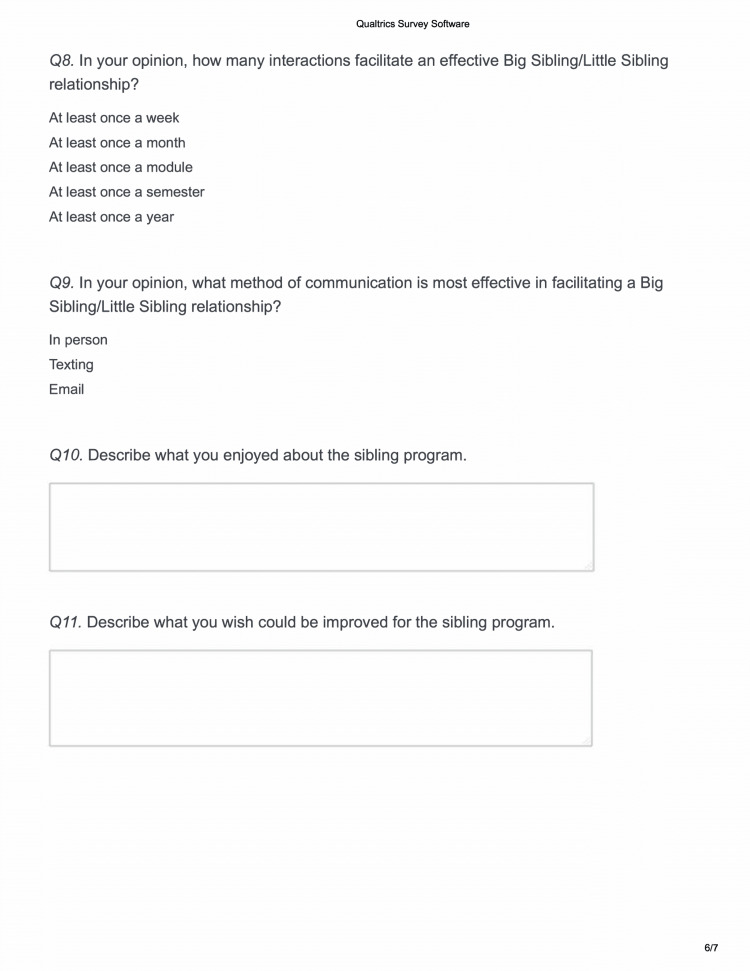
Six-month mentee evaluation survey (page 5)

## Appendices

Appendix A

Appendix B
